# Prevalence and management of syphilis at primary healthcare level in the Free State, South Africa

**DOI:** 10.4102/sajid.v40i1.724

**Published:** 2025-05-31

**Authors:** Olive P. Khaliq, Ahmad Jassen, Nomakhuwa E. Tabane, Jagidesa Moodley

**Affiliations:** 1 Department of Paediatrics and Child Health, Faculty of Health Sciences, School of Clinical Medicine, University of the Free State, Bloemfontein, South Africa; 2 Department of Obstetrics and Gynaecology, School of Medicine, University of KwaZulu-Natal, Durban, South Africa

**Keywords:** maternal syphilis, screening, management, pregnancy, congenital syphilis, newborns

## Abstract

**Background:**

Maternal syphilis (MS) is of special concern because of the risks of vertical transmission resulting in high rates of stillbirths and neonatal infections, especially in low- and middle-income countries (LMICs), such as South Africa (SA).

**Objectives:**

To assess the clinical management of MS at two primary healthcare clinics.

**Method:**

This was a retrospective evaluation of the antenatal records from 2020 to 2023 at two clinics in the Free State, SA. Demographic and clinical data, including MS mono rapid plasma reagin test and HIV status measured using the mono rapid HIV test, foetal and perinatal outcomes were collected.

**Results:**

668 records were reviewed. Fifteen tested (2.3%) positive for MS, but only 12/15 received complete treatment. Of the syphilis negative women, only 365 (55.3%) were retested. 28% of all pregnant women were HIV-positive. Four of the 15 (27%) women with MS had HIV, while 11 of the 15 (73%) of the HIV-negative pregnant women had syphilis. Among syphilis-exposed neonates, two had complications due to syphilis exposure and one had low birthweight despite maternal treatment.

**Conclusion:**

The prevalence of MS at the study sites was 2.3%. 44.7% of the women who tested negative for syphilis were not retested, and three women with syphilis did not receive complete treatment. Incomplete treatment of the mothers’ results in complications in syphilis-exposed neonates. There is an urgent need for continuing training for the nurses and midwives on antenatal screening and treatment protocols for MS at primary healthcare settings in the Free State, SA.

**Contribution:**

This work will benefit the community by alerting the Department of Health on the short comings found at antenatal care clinics, with the goal to improve the management of pregnant women.

## Introduction

The World Health Organisation (WHO) reported that 8 million people globally were infected with syphilis in 2022, with most infections occurring in sub-Saharan Africa (SSA) and Southern Asia.^1,2^ Syphilis, caused by the bacterium *Treponema pallidum* is transmitted through sexual contact, vertically from mother to child during pregnancy and through blood transfusions.^1^ Syphilis occurs in different stages; primary, secondary and tertiary syphilis, each with different signs and symptoms. However, in some cases, there are no signs or symptoms, referred to as latent, usually diagnosed when presenting with other illnesses or during pregnancy and childbirth.^3^

Ninety percent of all syphilis cases occur in low- and middle-income countries (LMICs).^4^ This staggering statistic highlights a significant public health challenge as untreated syphilis, especially during pregnancy can lead to serious adverse events for both the mother and her baby. Maternal syphilis (MS) is associated with complications such as stillbirths, preterm births, low birthweight, foetal growth restriction and congenital syphilis (CS).^5^ This underscores the urgent need for primary and secondary prevention by effective screening, treatment and education strategies, especially within antenatal care settings is required.

The National Institute for Communicable Diseases (NICD) reported troubling syphilis trends in pregnancy.^6^ In 2019, 2.6% of pregnant women in South Africa (SA) were diagnosed with syphilis, marking a concerning 30% increase from 2015.^6,7^ In addition, syphilis occurred in approximately 2.9% of all pregnancies in the SSA region in November 2018.^8^ Syphilis also increases the likelihood of infected individuals contracting other viral and bacterial infections, including HIV.^9^

The prevalence of HIV in SA is high, especially in pregnancy, raising significant public health concerns. In 2022, 25.3% of pregnant women were living with HIV in South Africa.^7^ These figures have increased since the 2020 report indicated a HIV prevalence of 22.9% in women aged 15–49 years.^10^ Various factors contribute to the high HIV prevalence, including socioeconomic challenges, limited access to healthcare and widespread misconceptions about HIV and its transmission.^11^ HIV is also associated with a higher risk of other sexually transmitted infections.^12^ The most common infections found in HIV-positive individuals are gonorrhoea, chlamydia, trichomonas vaginalis, herpes simplex virus type 2 and syphilis.^13^ HIV negative individuals may also get sexual transmitted infections (STIs), but their risk of infection is lower than HIV positive individuals.^14^ In addition, STIs increase the risk of acquiring HIV infection through inflammation by increasing the viral concentration and in the genital tract.^15^

The prevalence of MS and HIV-co-infection was 6% in the Gauteng province and 17% in Cape Town.^16^ Another study found that women living with HIV were more likely to acquire syphilis than women without HIV (odds ratio [OR]: 2.25, 95% confidence interval [CI]: 1.91–2.64).^17^

The Free State province has no recent published data on MS except from the annual South African Antenatal Survey Reports (2022). The only published article from the Free State was in 1994 on the prevalence of vaginal infections, syphilis and HIV in 483 women from the rural area and 475 women from the urban area. The women from the rural area were recruited from a farm, while the urban women were recruited from the township maps.^18^ The rates of syphilis and HIV infections were higher in urban than in rural settings. In rural settings, 12% had syphilis, while in the urban settings, 16% had the disease. The prevalence of HIV was 0.4% in rural settings and 1.5% in urban settings. Seventy-eight per cent of rural women had less than 6 years of education compared to 34% of urban women.^18^

Given the reported increasing trends of STIs, especially syphilis in SA,^19^ it is essential to investigate how midwives and nurses screen pregnant women for syphilis and other STIs. Assessing whether midwives and nurses adhere to established guidelines for the treatment and prevention of MS is crucial to decrease perinatal deaths, prevent CS and lower rates of other STIs. This study aimed to assess the management of syphilis at two primary healthcare clinics in the Free State province of SA.

Congenital syphilis is a substantial global health challenge, resulting in a range of adverse outcomes during the antenatal and perinatal periods. These outcomes include stillbirths, intrauterine foetal deaths, neonatal deaths, congenital infection-related morbidities such as gastrointestinal disorders and long-term disabilities, notably postnatal growth restriction.^20^ Globally, an estimated 661 000 cases of CS occur annually, with LMICs, particularly in SSA and South Asia, contributing the majority.^21^ In SA, the statistics regarding CS present a concerning situation where the notifications increased. One hundred and twenty-seven cases were reported in 2018, 269 cases in 2019 and 373 cases in 2020, rising from 13 to 37 cases per 100 000 live births.^22^

In 2007, the WHO initiated the Global Elimination of CS Initiative, aiming to reduce vertical transmission of syphilis.^23^ Despite these efforts, CS remains a significant public health issue, nearly two decades later.^23,24^ Currently, no specific diagnostic tests exist for CS, as a result, all live-born or stillborn babies born to mothers with syphilis should undergo thorough evaluations for congenital infection.^25^

This evaluation helped ensure that pregnant women received the necessary screening tests for STIs and interventions during antenatal visits to protect their health, that of their unborn babies and their family or partner. By understanding the clinical screening practices at these clinics, areas for improvement were identified to enhance the overall quality of care provided to pregnant women staying at SA.

This study’s primary healthcare facilities used the Guidelines for Maternity Care in South Africa, 2016, stating that all pregnant women should undergo a syphilis screening test before 20 weeks of gestation. If negative, the test should be repeated at 32–34 weeks of gestation. The standard treatment is benzathine penicillin, irrespective of the titre.^26^ The midwives at the clinics reported that they advise the patients who tested positive to notify their partners and offer referral cards for the clinics of their choice. However, there is no evidence that the partners get notified or seek treatment.

## Research methods and design

A cross-sectional retrospective evaluation of maternal records was done at two primary healthcare facilities from 2020 to 2023 in the Free State. The study sites were selected by location, size and number of antenatal patients attending the clinics. The study clinics selected had large numbers of antenatal attendees daily and were considered representative of the population in the Free State.

Ethical and provincial health authority permissions were obtained prior to the initiation of the study (UFS-HSD2022/1399/2803-0001). Maternal demographic and clinical data, syphilis and HIV status, as well as foetal and perinatal outcomes were collected. All pregnant women who visited the antenatal clinic from January 2020 to May 2023 were included, regardless of their syphilis or HIV status.

All nurses and midwives at the primary healthcare clinics follow maternal health guidelines provided by the National Department of Health as follows: The standard antenatal care procedures for screening syphilis status was initiated using test for the rapid plasmin reagin (RPR), RPR tires and *T. pallidum* Haemagglutination assay (TPHA) at the first antenatal visit. The RPR measures whether the pregnant woman had syphilis previously or has active disease. Rapid plasmin reagin titres of 1:8 and greater were indicative of active disease, while titres of 1:4 or less usually indicate previous infection. The TPHA, a more sensitive test, is used to diagnose secondary and tertiary syphilis.^27^

The South African sexually transmitted infection guidelines state that all pregnant women should be tested for syphilis and HIV at their first antenatal visit.^28^ A repeat test for syphilis is done at 32 weeks for those who tested negative in the first trimester. For a positive test, a patient receives three doses of benzathine penicillin (2.4 MU), intramuscular, once weekly for 3 weeks. A rapid test is also used to screen for HIV.^27^ Syphilis-exposed children are given benzathine penicillin (50 000U/kg) intramuscularly, if symptomatic.^27^

### Statistical analysis

All the data points were analysed descriptively. The categorical variables were described using frequencies and percentages. The continuous variables were summarised using mean and standard deviation or median and interquartile range. The association between categorical variables was explored using the Chi-square or Fisher’s exact test. The variables with a statistically significant difference show *p* < 0.05.

### Ethical considerations

Ethical clearance to conduct this study was obtained from the University of the Free State, Health Sciences Research Ethics Community HSREC (reference no.: UFS-HSD2022/1399/2803-0001).

## Results

The study comprised 668 maternity records from January 2020 to December 2023 at two primary healthcare clinics. Most pregnant women were between the ages of 19 and 35 years (91.9%), followed by those aged 18 years and below (5.7%). Lastly, the ≥36 age group was the lowest with 2.4%. The median age was 25 and the interquartile ranges were 21–30 (Q1–Q3). 66.2% of the women had gravidity between 2 and 5, while 67.3% had a parity of 1–5. Additionally, there was a significant association between parity and age (*p* < 0.0001).

In their previous pregnancies, intrauterine foetal deaths occurred in 6.3% of the study population, two (0.4%) had preterm births with no significant association to age (*p* = 0.0862) and seven (1%) had a termination of pregnancy by choice (Table 1).

**TABLE 1:** Maternal demographic data and clinical characteristics.

Maternal characteristics	Median	Interquartile range (^1^Q1–^2^Q3)	Minimum–maximum ages	Frequency	Percentage	*P*
**Maternal age (years)**	25	21–30	15–43	-	-	-
≤ 18	-	-	-	38	5.7	-
19–35	-	-	-	614	91.9	-
≥ 36	-	-	-	16	2.4	-
**Obstetric history**						
Gravidity	-	-	-	-	-	-
01	-	-	-	210	31.4	-
02–05	-	-	-	456	66.2	-
≥ 06	-	-	-	1	0.1	-
**Not recorded**	-	-	-	1	0.1	-
Parity	-	-	-			< 0.0001
00	-	-	-	225	33.7	-
01–05	-	-	-	442	67.3	-
Not recorded	-	-	-	1	0.2	-
**Previous pregnancy history**						
Intrauterine foetal death	-	-	-	-	-	-
Yes	-	-	-	42	6.3	-
Preterm birth	-	-	-	-	-	0.0862
Yes	-	-	-	2	0.4	-
Choice of termination of pregnancy (CTOP)	-	-	-	7	1.0	-
**Antenatal care**						
Gestational age at first antenatal visit (weeks)						
First trimester	-	-	-	43	6.4	-
Second trimester	-	-	-	471	70.5	-
Third trimester	-	-	-	150	22.5	-
Labour	-	-	-	4	0.6	-

Q1, quartile 1; Q3, quartile 3.

### Antenatal care

In the first trimester 43 (6.4%), in the second trimester 471 (70.5%), in the third trimester 150 (22.5%) and during labour 4 (0.6%) of the pregnant women initiated their first antennal visits (Table 1).

The initial syphilis test was performed in 11 women (1.6%) in their first trimester, 587 (87.9%) in their second trimester and 62 (9.3%) in their third trimester. Eight women (1.2%) were not tested for syphilis at their first visit.

The RPR test used during the initial visit in 647 (96.9%), RPR titres test was performed in three women (1.9%). One woman (0.6%) with previous primary syphilis infection had a positive TPHA and nine syphilis tests (1.3%) were not specified. For those who tested positive at the initial syphilis test, none received a confirmatory TPHA test (Table 2).

**TABLE 2:** The management of maternal syphilis (*N* = 668).

Syphilis screening test	Frequency	Percentage
**Gestational age at which first test was done**		
First trimester	11	1.6
Second trimester	587	87.9
Third trimester	62	9.3
Not tested	8	1.2
**Syphilis test used for screening (initial) (*n* = 660)**		
Mono rapid plasmin reagin test	647	96.9
RPR titres	3	1.9
*Treponema pallidum* haemagglutination assay (TPHA)	1	0.6
Not specified	9	1.3
**Maternal syphilis status (at first antenatal visit test)**		
Reactive	15	2.3
Non-reactive	645	97.7
Not tested	8	1.2
**Was a syphilis repeat test done?**		
Yes	365	55.3
No	295	44.7
**Gestational age at which repeat test was done (*n* = 365)**		
First trimester (once)	113	16.9
Third trimester (once)	142	21.3
In Labour (once)	1	0.1
First and third trimester (twice)	97	14.5
Third trimester, in labour (twice)	3	0.4
First (once) and third trimester (twice)	3	0.4
Third trimester (twice), in labour	5	0.7
First, second, third trimesters and (four times)	1	0.1
**Gestational age at which syphilis treatment was initiated (*n* = 15)**		
First trimester	7	46.7
Second trimester	6	40
Third trimester	1	6.7
Not treated	1	6.7
**Was there a delay in treatment?**		
Yes	3	20
No	11	73.3
**Treatment of syphilis infected (penicillin doses) before delivery**		
One dose	1	6.6
Two doses	1	6.6
Three doses	12	80
**Was penicillin given 28 days before childbirth**		
Yes	14	93.3
No	1	6.6
**Were there any allergic reactions to penicillin?**		
Yes	0	0
No	14	93.3
**Follow-up treatment**		
Yes	6	40
No	9	60
**Test done following treatment**		
RPR titres	5	33.3
Treponemal	1	6.6
**Intervals at which tests were done following treatment**		
6 weeks	3	20
12 weeks	2	13.3
Unknown	1	6.3
**Baby outcomes**		
Baby weight		
1000 g – 1499 g	1	0.1
1500 g – 2499 g	43	6.4
> 2500 g	624	93.4
**Congenital abnormalities**		
Dysmorphic	1	0.1
Abdomen hepatosplenomegaly	1	0.1

TPHA, *Treponema pallidum* haemagglutination assay; RPR, rapid plasma reagin.

Of the 668 women in the study, 660 (98.8%) were tested for syphilis; 15 (2.3%) tested positive, while 645 (97.7%) tested negative. For the latter, only 365 (55.3%) were retested. Thirty per cent (109) were tested more than twice. Rapid plasmin reagin titre results post-treatment were not documented (Table 2).

### HIV testing

Twenty-eight per cent (187) women tested positive for HIV. A repeat HIV test was performed in 333 (49.8%) of previously negative women, with 1 (0.15%) seroconverting (Table 3).

**TABLE 3:** HIV prevalence and syphilis co-infection.

Variables	Frequency	Percentage
**HIV status (*n* = 668)**		
Positive	187	28
Negative	481	72
HIV repeat test	333	49.8
**Seroconversion**		
Negative to positive after retest	1	0.15
**HIV treatment (*n* = 187)**		
On treatment	157	84
Not on treatment	30	16
**Syphilis-HIV co-infection (*n* = 15)**		
HIV positive, syphilis positive	4	27
HIV positive, syphilis negative	11	73

### Syphilis-HIV co-infection

Of the 15 pregnant women diagnosed with syphilis, four (27%) were HIV positive and 11 (73%) were HIV negative (Table 3).

## Treatment

### Syphilis

Of the 15 pregnant women with syphilis, only 12 received three doses of intramuscular benzathine penicillin during the first and second trimester. There were delays in treatment for three women – the reason for one was because there was a shortage of sterile water for the penicillin injection. The reasons for the other two delays were not recorded. No one was allergic to penicillin. Follow-up investigations were only done in six of the 15 syphilis infected women to evaluate whether the RPR titres decreased. No further doses of penicillin were recorded. Syphilis retesting following treatment was done in the six women to monitor treatment response. The intervals at which the tests were done were 6 weeks in three women and 12 weeks in two women.

### HIV

Of the 187 (28%) pregnant women with HIV, 157 (84%) were on treatment, while 30 (16%) were not on treatment.

### Neonatal outcomes

Neonatal outcomes based on weight revealed that 43 (6.4%) babies had low birthweight, while 624 (93.4%) had normal birth weight. Additionally, 1 (0.1%) exhibited hepatosplenomegaly and 1 (0.1%) showed signs of dysmorphism.

The birth outcomes of syphilis-exposed neonates indicated that 14 had normal birth weights, 13 of whose mothers were treated, while one was born to an untreated woman. One neonate had a low birthweight despite the woman initiating antenatal care early and receiving three doses of penicillin, however, the interval between treatment and childbirth was less than 28 days. No follow-up testing was conducted after treatment. Information regarding the testing and treatment of exposed infants was unavailable in the maternity case records. Of the HIV-exposed babies, 20 (10.7%) had low birthweight. Of these, seven were born from HIV-positive, untreated women. One baby from a mother on antiretrovirals had very low birthweight and hepatosplenomegaly. All the babies with both syphilis and HIV exposure (*n* = 4) were term with a birthweight >2500 g.

## Discussion

### Main findings and interpretations

Nearly two-thirds (471, 70.5%) of the women in the current study initiated antenatal care during the second trimester. These women are referred to as *late bookers* since antenatal visits are recommended after the first menstrual period is missed. According to the guidelines for maternity care in South Africa, 2016, women should visit the antenatal clinic as soon as they suspect pregnancy or soon after their first missed period. In the first antenatal visit, screening investigations should be performed. These include HIV, syphilis, TB, Haemoglobin, Urinary dipstick (protein and glucose, at each visit) and Rhesus blood group.^26^

Not all women were tested for syphilis; 660 (98.8%) of 668 women had syphilis screening tests. The results of this study are in keeping with an article published in 2019 on syphilis screening coverage and positivity by HIV treatment status among SA pregnant women.^10^ The findings showed that among the 35 900 women studied, 96.3% were screened for syphilis, with a variation among the provinces, with the highest screening coverage of 99.1% in the Free State, similar to 97.7% in the current study and the lowest being the Limpopo province at 90.8%.^17^

Of the 660 women tested, 15 (2.3%) were positive for syphilis. Of these women, 14 (93%) received treatment. These results differ from the 2008–2011 and 2015 South African National syphilis surveillance report for the Free State, which reported a prevalence of 1.9% and 4.6%, respectively.^29^ This could be because our study only included two community health centres.

In the current study, a repeat test was only done in 55.3% of the women who initially tested negative. The STIs guidelines of SA (2018) state that all pregnant women must be tested for syphilis at their first antenatal visit with negative tests repeated at 32–34 weeks gestation. Some women who initially test negative at the first antenatal visit may not have developed an immune response or become infected during the pregnancy. It is therefore recommended that screening for MS be repeated at varying gestational ages and at the time of childbirth.^27^ In addition, the new guideline for vertical transmission prevention of communicable infections of South Africa (NdoH), 2023 states that syphilis retesting should be done 4 weekly intervals, during labour or delivery and at the time of intrauterine death diagnosis.^30^

Three doses of benzathine penicillin were given to 12 pregnant women. Two women did not receive three doses in the current study; the reasons not stated in the maternal file. However, all 14 women received treatment before childbirth. The STI guidelines from 2018 recommend that pregnant women with syphilis should receive benzathine penicillin once a week for 3 weeks (3 doses). Our results are similar to those reported in KwaZulu-Natal, SA, where the three-dose compliance was assessed at an antenatal care clinic. This was a retrospective study conducted on 18 128 maternal records between 2001 and 2002. The results revealed that 188 (1.04%) of the women had syphilis. In terms of treatment, 15.9% of the women were not treated, 5.8% who were treated only received one dose and 13.2% received two doses.^31^

Furthermore, the current study demonstrated that of the 15 women who tested positive for syphilis, 27% were also HIV-positive. These results were contradictory to other studies as most claim that syphilis infection predisposes women to HIV.^32,33,34^ A systematic review and meta-analysis from China reported on the effect of syphilis on HIV acquisition and found that the incidence of HIV had a two-fold increase in individuals with syphilis.^32^ Another study in the New York City, US, investigated HIV diagnosis following syphilis infection in men and reported that 15.1% of men with syphilis acquired HIV. A more interesting finding in this study was that the incidence of HIV was significantly higher in men with secondary syphilis (4.10%) compared to men with primary syphilis (2.6%) (*p* < 0.0001).^35^ In SA, a study published in 2020 on the prevalence and risk factors associated with HIV and syphilis co-infection reported that 3.1% (87/2818) of people living with HIV had serological evidence of syphilis and 11 of the 87 had an RPR titre of >1:8.^36^

This study found an HIV prevalence of 28% with only one (0.15%) seroconversion. These results are lower than Hoque et al., who reported a seroconversion of 4% in pregnant women of South Africa in 2021.^19^ In another study conducted in 2022, the seroconversion of HIV in pregnant women decreased from 4% to 3.1% before delivery and 3.2% post-delivery.^37^ These discrepancies may be due to the higher prevalence of HIV infection in these studies compared to the current study (44.3% and 44.7% versus 28%).

In this study, one woman did not receive treatment due to the unavailability of sterile water for the benzathine penicillin injection. South Africa is a low- and middle-income country burdened with a high prevalence of STIs such as HIV and syphilis. In addition, the country suffers from a deficit of medical resources, resulting in delayed treatment or no treatment at all. Similar evidence was reported in the Gauteng province of South Africa where challenges in antenatal care service delivery in semi-urban healthcare facilities were explored.^38^ The study highlighted factors such as insufficient medical supplies, challenges related to the COVID-19 pandemic that resulted in a shortage of medical supplies and a shortage of staff, among others.^38^ These challenges are not only limited to SA; a study in the Netherlands also described similar challenges in antenatal care settings. Medical products and machinery, health service delivery and health financing were mentioned, among others.^39^ Therefore, the lack of medical resources seems to be a common challenge in high-income and low- to middle-income countries.

Of the 15 syphilis-exposed babies, one (0.1%) had a low birthweight. Our findings differ from a retrospective, cross-sectional study of 306 cases of syphilis in pregnancy in Brazil, by Padovani et al., that showed a prevalence ratio of 60% higher for low birthweight (OR: 1.6, 95% CI: 1.14–2.28).^40^ Similarly, a longitudinal retrospective study of 155 214 (8.7/1000) live births exposed to syphilis showed that MS increases the OR of low birthweight babies (OR: 1.53, 95% CI: 1.51–1.56).^41^ Furthermore, one (0.1%) of the syphilis exposed babies presented at birth with hepatosplenomegaly and one (0.1%) with dysmorphic features. The low numbers may be because syphilis was detected earlier during pregnancy.

### Strengths and limitations

The strengths of this study are that it was conducted in primary healthcare facilities in rural settings. Furthermore, the study was conducted over a 3-year period and had a sufficient sample size.

The study had limitations, as there were neither records of attempts made to contact the woman’s husband or partner, nor any discussions with the antenatal attendee regarding referral of the partner for investigation and treatment, if appropriate.

### Recommendations

There is a need for frequent monitoring to ensure that the midwives and nurses are following the guidelines for syphilis testing, ensuring that the treatment of maternal and neonatal syphilis and contact tracing of the male partner is adequately done.

Syphilis retesting for women who tested negative should be done regularly at different gestational ages and before delivery as infection may occur at any point during pregnancy. Nurses and midwives should receive training on syphilis testing. The health facility management and district clinical specialists should perform regular audits to ensure availability of sufficient testing kits and treatment through frequent and regular audits. Awareness of other sexually transmitted diseases besides HIV should be emphasised among all pregnant women. Education on the importance of early antenatal visits as soon as the first menstrual period is missed to avoid late diagnosis of diseases that could lead to complications.

## Conclusion

Repeat tests were not performed in 44.7% of the pregnant women managed at the clinics, 3 of the syphilis infected women did not receive complete treatment. Exposed babies were born with congenital abnormalities and low birth weight. There is an urgent need to improve the syphilis management program in clinics by ensuring the SA guidelines are implemented by regular training of the professional nurses and clinic managers.
